# CFTR Modulators Therapy Efficacy in Reducing Cystic Fibrosis (CF) Exacerbation and Improving Selected Spirometry Parameters: A Real-Life Study in a Single-Centre Polish Population

**DOI:** 10.3390/jcm13154491

**Published:** 2024-07-31

**Authors:** Hanna M. Winiarska, Daria Springer, Filip Wojtaś, Ewa Wysocka, Szczepan Cofta

**Affiliations:** 1 Department of Pulmonology, Allergology and Pulmonary Oncology, Poznan University of Medical Sciences, 84 Szamarzewskiego St, 60-569 Poznań, Poland; dspringer@ump.edu.pl (D.S.); fwojtas@ump.edu.pl (F.W.); scofta@ump.edu.pl (S.C.); 2 Chair and Department of Laboratory Diagnostics, Poznan University of Medical Sciences, 84 Szamarzewskiego St, 60-569 Poznań, Poland; ewysocka@ump.edu.pl

**Keywords:** cystic fibrosis, CFTR modulators, spirometry, exacerbations

## Abstract

**Background/Objectives:** Cystic fibrosis is a genetically determined disease that significantly influences and shortens life. Treatment with CFTR modulators (CFTR-T) is a new hope for patients. It can change the predictive values of a poor prognosis (e.g., exacerbation rate and FEV1 value). The aim of the study was to analyse exacerbation incidence and spirometry data before and after one year (+/− 2 weeks) of CFTR-T in 85 CF patients at the CF Centre in Poznań. To our knowledge, this is the first analysis of CFTR-T efficiency in the Central–Eastern Europe population. **Methods**: We retrospectively analysed the spirometry and exacerbation data of 85 CF adult patients (both men and women), who in the middle of 2022 began treatment with CFTR modulators. **Results**: The one-year ratio of hospitalisation caused by severe exacerbations lowered from 1.25 to 0.21 per patient per year. We also saw a 66% decline in ambulatory exacerbations. The median FEV1% increased by 9.60% in absolute values and by 460 mL. Even in the group with very severe obstruction (FEV1 < 35%), there was an increase in median FEV1% of 5.9 in absolute values. We also proved the increase in FVC% (median 17.10% in absolute value and 600 mL) in the study group. **Conclusions**: After one year of treatment, an impressive improvement was observed in two important predictive values of poor prognosis: exacerbation rate and FEV1 values. Further observation is needed to determine how long the improvement will be present and its influence on quality of life and life expectancy.

## 1. Introduction

Cystic fibrosis (CF) is a genetically autosomal recessive disease associated with chloride channel pathology. Over 1800 cystic fibrosis transmembrane regulator (CFTR) mutations associated with CF disease were identified. The type of mutation determines protein production, activity, and cell membrane presence, which causes differences between the clinical manifestations of the disease and treatment possibilities [1].

The treatment of CF disease should focus on gene therapy or on repairing the pathological production, maturation, transportation, conductance, and stability of the CFTR protein.

The success of modulators-based therapy—CFTR-T (elexacaftor, tezacaftor, and ivacaftor) depends on the kind of mutation and the stage of organopathy caused by CF. There are many more factors influencing success: chronic infection with multidrug-resistant bacteria, liver function, proper lipid absorption (appropriately selected dose of pancreatic enzymes), and last but not least, patient-dependent factors: the correctness and regularity in medication use (modulators and other drugs), diet, physiotherapy appropriate to the advancement of the disease, and general health-promoting and health-harming behaviours. Thus, we suspect that real-life data may differ from the results obtained in drug registration studies.

The natural history of CF patients involves progressive lung disease, leading to chronic respiratory failure. Improving respiratory system functioning is the most crucial goal of all CF therapies. X-ray changes, lung function tests, the microbiology of sputum (especially for *P. aeruginosa* infections), the rate of worsening, body mass index (BMI), and weight loss can all help predict if the respiratory system is sufficient [2].

Respiratory pathology was the most common primary cause of death in the CF population before CFTR modulator treatment, responsible for 50.4% of deaths [3]. In 2022 (so after the beginning of CFTR modulator treatment), the rate was 38.3% [4]. Thus, we can see the underlying meaning of respiratory diseases and lung-transplant complications as a reason for death in the CF population.

Exacerbations are associated with lung function decline [3]. In 2008, 38% of adult patients with CF in the Cystic Fibrosis Foundation Patient Registry were treated with intravenous antibiotics for at least one pulmonary exacerbation [4]. Patients having one or more CF-related hospitalisations during a year have a significantly higher 8-year mortality and worse lung function [5]. Some data show that the 1-year mortality rate among patients with severe CF exacerbations was between 9% (non-ICU treatment) and 48% (ICU treatment) [6]. The need for rehospitalisation because of severe exacerbations during one year was 34% (ICU patients) and 26% (non-ICU patients) [6].

A large cohort study based on 8479 pulmonary exacerbations showed that almost 25% of patients failed to recover to baseline FEV1 in three months after exacerbation. Of those who did not reach the baseline after three months, 75% did not reach it six months after treatment, and 58% did not recover even 12 months after exacerbation [4].

Thus, exacerbation rates greatly influence FEV1 decline, and, as a result, the mortality rate in CF patients. On the other hand, the low FEV1% value is a risk factor for a shorter time without a new exacerbation [7]. Those two factors are strongly associated.

The natural history of cystic fibrosis is related to the constant reduction in FEV1% [8]. The decline in FEV1 strongly predicts mortality in the CF population [9]. The FEV1, measured as a percentage of the predicted value, is the best generally available measure for assessing CF lung disease [10]. It defines a disease stage and helps to make therapeutic decisions, and its level is also one of the lung transplant indicators [11]. An FEV1% value lower than 30% is considered a general lung transplant indicator—the risk of death without the operation in those patients in the next two years is higher than 50% [11].

Treatment improving spirometry parameters in CF, especially the FEV1%, can prolong life duration, quality of life, and the need for a lung transplant.

The aim of the study was to determine the influence of treatment by CFTR modulators on the number of exacerbations, FEV-1, and FVC changes (as the most important predictors of overall survival) during the first year after the beginning of treatment of adult patients with cystic fibrosis in the CF Centre in Poznań University Hospital.

## 2. Materials and Methods

After receiving the Bioethics Committee’s consent, we retrospectively analysed ambulatory visits and hospitalisations in our CF centre for 85 adult patients who began treatment with CFTR modulators (CFTR-T) in 2022. We analysed the data of patients with exacerbations (divided into hospital and ambulatory treatment) and spirometry results (done in a stable moment of the disease) four years before CFTR-T began, in 2022 (just before starting the therapy) and in 2023 (after one year of treatment). The initial group in our centre grew every year (2018-51, 2019-60, 2020-65, 2021-75, 2022-85, 2023-85). Table 1 shows the baseline patient characteristics.

Our study defined CF exacerbations (CF-Ex) based on the EURO Care CF Working Group definition, which improved the Fuchs criteria [12].

All spirometry tests were made following the 2019 American Thoracic Society and European Thoracic Society Technical Statement [13] on the Viasys Healthcare MasterScreen IOS Spirometer.

We analysed the data of spirometry results:

- FEV1 as a percent of predicted value(FEV1%) and measured in litres (FEV1-L);
- FVC as a % of predictive value (FVC%) and measured in litres (FVC-L).

We also divided patients into smaller groups based on ERS/ATS guidelines [14] to:

- FEV1% > 70 (mild obstruction);
- FEV1% 60–69 (moderate obstruction);
- FEV1% 50–59 (moderately severe obstruction);
- FEV1% 35–50 (severe obstruction);
- FEV1% < 35 (very severe obstruction).

Then, we compared the FEV1% change in those groups between 2022 and 2023. We also compared the exacerbation rate between 2022 and 2023 in groups mentioned above. The results are shown in Table 1.

Statistical calculations were performed using the Statistica 13.0v program (StatSoft, New York, NY, USA). In all the tests, *p* < 0.05 was taken as an indicator of statistical significance.

We used the Shapiro–Wilk test to find the characteristics of the parameter distribution in the studied population. Due to the lack of confirmation of a normal distribution, non-parametric tests were used for subsequent statistical analyses. In descriptive statistics, data were presented as the median and interquartile range.

We used the Wilcoxon signed-rank test to compare the spirometry results for each patient between 2022 and 2023. The last spirometry before treatment begins and ends after one year of treatment. We also compared the exacerbation rate between subsequent years by using graphs.

## 3. Results

### 3.1. Exacerbations

**Figure 1:**
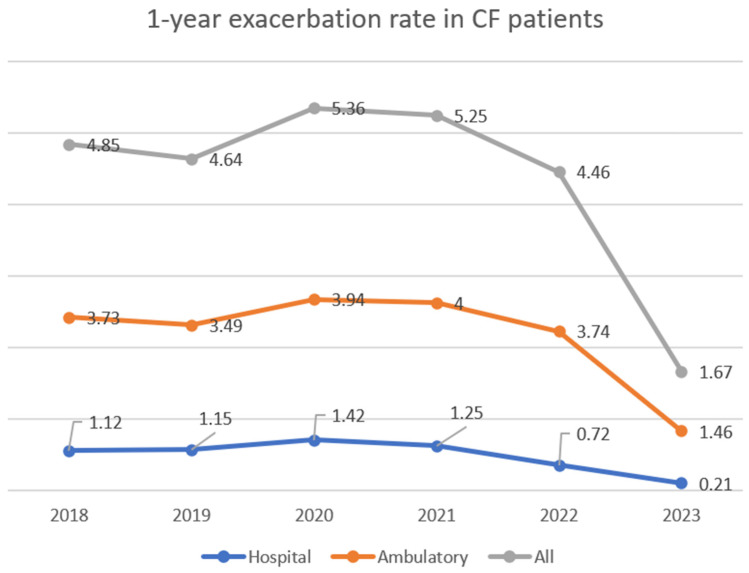
The exacerbation rates (exacerbation/patient/year) in CF patients in the analysed years, with the specification of the hospitalisation and ambulatory characteristics.

Because there is a difference between the number of patients in subsequent years, we counted the one-year ratio of hospitalisation caused by exacerbations (ratio = quantity of exacerbations/group quantity). We compared 2021, before the beginning of treatment, and 2023, after 1-year treatment. There was an 83.2% reduction in the hospitalisation ratio. We used the analogical ratio to analyse the ambulatory treatment of exacerbations: there was a 66% decline in the exacerbation ratio one year after the beginning of treatment compared to 2021. The data for all kinds of exacerbations are like those previously shown. We can see an increase in all exacerbations between 2018 and 2021, with a considerable decline (63.58%) in 2023. The exacerbation ratio dropped from 5.20 to 1.67 (67.88%) between 2021 and 2023. Figure 1 displays all the data.

### 3.2. Spirometry

Table 2 shows the spirometry data as median values and interquartile ranges. Additionally, we compared the data from 2022 and 2023 with the Wilcoxon signed-rank test.

The median FEV-1 value as a percent of predictive value increased by 9.26 and 0.46 litres. The median FVC increased by 17.1% of predictive value and 0.06 litres.

It is essential to determine if the success in spirometry parameters elevation refers to all obstruction severity groups. Table 3 shows the spirometry data in 2022 and 2023 in the study group, divided into smaller ones based on obturation severity. We showed the data as a median and interquartile range in 2022 and 2023. All differences were significantly relevant; we used the Wilcoxon signed-rank test. The biggest FEV1% absolute value difference was seen in the moderate obturation group—14.4%. We can also see the change in a group with severe obturation: a 5.9% difference in absolute value.

**Figure 2:**
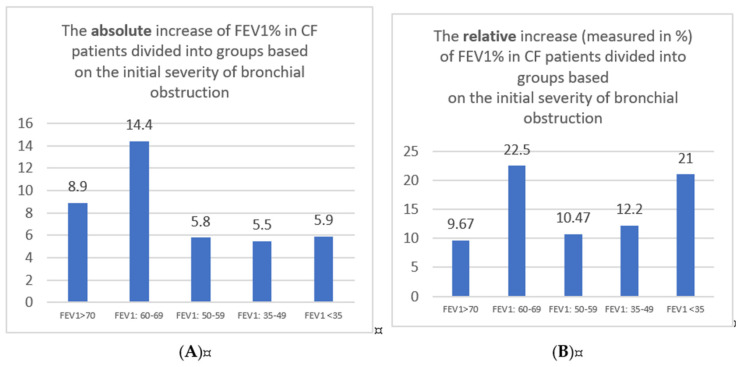
(**A**) The absolute FEV1% median value increase between 2022 and 2023 in subsequent groups of analysed CF patients. (**B**) The relative increase in FEV1% median value between 2022 and 2023 in subsequent groups of analysed CF patients.

Figure 2A displays the data.

It is difficult to compare the absolute values of FEV1% changes. For example, the exact FEV1% absolute value change in FEV1 > 70 groups will be less important than in the FEV1% < 35 groups. Therefore, we analysed the relative increase in median FEV1% in subsequent groups. Figure 2B displays the information.

The most significant relative change was observed in the group with moderate obstruction: a 22.5% relative increase in FEV1%. Surprisingly, the second group with the best results is the group with very severe obturation: a 21% relative increase. These data prove that even the most severe group with CF benefits from CFTR-T. What is interesting is that there is a relatively weaker improvement in groups with moderately severe and severe groups, and it needs further investigation.

## 4. Discussion

### 4.1. Exacerbations

CF is a multiorgan, severe, progressive disease. The modulator treatment is a hope for life extension and better functioning of the lungs. Because the treatment is relatively new, we are still waiting for large population-based data with the real-life efficiency of ETI treatment. We can find some small population studies and case reports, but the results are inconsistent. The differences can arise from the kind of population, general healthcare in a region, and non-medical factors influencing compliance. That is why our work, based on 85 patients, is so important. It is the first study based on the part of the Polish CF population evaluating the influence of CFTR-T on exacerbations and spirometry results one year after the beginning of treatment.

In a registration study of elexacaftor, tezacaftor, and ivacaftor number 445–102 [12], the exacerbation rate decreased from 113 (0.56 per patient per year) in the placebo control to 41 (0.02) in the ETI group. In our study, the exacerbation rate drops from 5.20 per patient per year to 1.64 per patient per year, so the data are even more promising than the registration study.

In the study of Gor et al. (196 participants), the number of exacerbations was reduced after two years of therapy. Patients not treated by ETI had a 4.48 times higher risk of exacerbation compared to ETI-treated patients [13].

The 2023 study summarises the effects of ETI on many general symptoms in the CF population with at least one F508 del allele in Germany—it included about 2600 participants. The exacerbations decreased by 75% one year after treatment began. Our data show a 67.88% decline in all exacerbations and a much more impressive decline in severe exacerbations treated in the hospital: the reduction was about 80.85%. Our study is much smaller, but the data are also promising.

On the other hand, another study based on the German population, with about 5000 participants, shows only 42% of exacerbation reduction. The authors suggest that the specificity of pandemic time, with isolation and hyper-hygienic behaviours, could influence the “pre-treatment” data and—as a result—lower the difference between “pre” and “post” exacerbations [14].

A study by Sutharsan et al. [15] provides data on reducing the number of hospitalisation-needing exacerbations. Before treatment began in a study population, the annual rate of hospital-required exacerbations was 1.41, and after one year, this rate fell to zero [15].

There is also a case report about a pregnant woman continuing ETI treatment. Despite the history of this woman, with many exacerbations during previous pregnancies, the one on ETI treatment was progressing without any exacerbation, and she delivered a healthy child vaginally [16].

### 4.2. Spirometry

In phase III of the ETI clinical trial, registration study number 445–102, the FEV1% growth was 13.9% after 24 weeks of treatment (13.5% in the first four weeks) [12].

Our study proved the elevation of FEV1% after one year of treatment with ETI. The absolute value of FEV1% increased by 9.2%.

Some studies were like ours but with different observation periods. In a small study of 38 adult patients presented by M. Wollsching-Strobel et al., the FEV1 percentage increased by 10.7% and FVC by 9.6% after 12 weeks of treatment [17]. In the other study (196 participants), the mean FEV1 improved after two years of ETI therapy at 10.5% (in absolute values) [13].

There are other ways to show the changes in the FEV1% value. Following ATS/ERS 2021, the FEV1 can be interpreted using a Z-score. In a small 5-month study, the Z-score FEV1% increased by 1.05 after five months of ETI therapy [18].

Tim Lee and coworkers conducted an interesting study. After stabilising FEV1 in patients receiving ETI therapy, they compared the annual FEV1% decline in the ETI group and the matched group without ETI treatment. While in the group without ETI treatment, the FEV1% lowered to 1.85–2.08% annually (depending on the genotype), in the group with ETI treatment, the FEV1 value elevated to 0.32–0.74% annually. The study lasted two years. They proved that ETI therapy was the first CFTR modulator therapy shown to halt lung function decline over an extended period [19].

The effectiveness of the modulator therapy in FEV1% improvement can also depend on baseline lung disease. Determining if the ETI treatment has similar outcomes in all CF severity groups is vital. In our study, there was an improvement in a group with all CF patients and groups with different severity diseases. The biggest increase in FEV1 was observed in the group with moderate obstruction (Fev1 60–69%)—the absolute increase of FEV1 was about 14.4%. There are some studies analysing the FEV1 absolute change. The study based on 34 patients showed that the FEV1% improved in all patients, but the most remarkable improvement was noticed in patients with baseline FEV1 50–70% [20]. According to our knowledge, our study is the first one to compare the relative improvement in FEV1. It is an important parameter: we cannot suspect a considerable increase in FEV1% in almost healthy patients—those with basal FEV1 > 70%. On the other hand, patients with very severe diseases can experience clinical improvement even with minor changes in the FEV1% in absolute values (but significant changes in relative ones). Our study also shows that patients with moderately severe and severe obstruction do not reach as significant a change in FEV1 as patients with very severe obstruction. There is a need to do further studies to determine the reason for those results.

Among the patients with a severe stage of obstruction (baseline FEV1 < 40%), the absolute increase in FEV1 after 12 months of ETI treatment was 8.9% [21]. However, the group was small and consisted of only 20 patients. In our study (also a very small group of 12 participants), the absolute increase in FEV1 in the very severe stage of the obstruction was about 5.9%.

On the other hand, patients with FEV1> 70% also improved in FEV1—at 7% after 12 months of treatment [22]. In our study, those patients reached 8.9% higher values than before the treatment.

The single study (50 participants) in Missouri proved that after two years of treatment, there was an improvement in all spirometry parameters, like in our study: FEV1%, FVC%, FEV1 L, and FVC L [23], which makes our study even more reliable. In our study, the improvement was also present in the FVC value (in percent predictive and absolute values) [23].

## 5. Conclusions

The modulator treatment of CF is an entirely new kind of therapy. The life-shortening disease, with a massive quality of life impairment, finally has a chance to become one of many chronic diseases, slightly affecting the patient’s life. The promising data from registration studies should be compared with real-life treatment results. It is too early to discuss CF morbidity in the era of modulator treatment, but we can see the improvement in the well-known risk factor for poor prognosis.

In our study, all participants were adults, which means they had many disease complications, especially in airway anatomy. It is even harder to expect improvement in destroyed lungs, with bronchiectasis containing mucus and many antibiotic-resistant microorganisms.

We suggested a real-life study to see excellent drug efficiency even after considering patients’ heterogeneity, comorbidities, and health-promoting and health-harmful behaviours in real life.

In our research, we analysed two factors of poor prognosis: exacerbation rate and spirometry results (especially FEV1%), and we proved that both can be improved even in the adult population.

After one year of treatment, impressive improvement was observed in two important predictive values of poor prognosis: exacerbation rate and FEV1 values. Further observation is needed to determine how long the improvement will be present and its influence on quality of life and life expectancy.

We also confirmed that the population with the most severe obstruction can also improve FEV1% parameters; if we consider the relative FEV1% increase, this group achieves vast benefits.

## Figures and Tables

**Table 1:** Characteristics of CF patients involved in the study.

	Number	Age (Median and Interquartile Range)	F508del Homozygotes	F508del Heterozygotes	FEV1 >70%	FEV1 60–69%	FEV1 50–59%	FEV1 35–49%	FEV1 <35%
Female	50	25 (22–27)	29	21	10	10	9	12	9
Male	35	28 (21–32)	15	20	15	5	4	6	5
All	85	27 (22–30)	44	41	25	15	13	18	14
All—exacerbation rate 2022					3.21	3.29	3.90	4.17	3.69
All—exacerbation rate 2023					0.16	0.14	0.30	0.47	0.75
Ambulatory exacerbation rate 2022					4.11	3.50	3.73	4.53	3.00
Ambulatory exacerbation rate 2023					1.89	0.86	0.91	1.89	1.69
Hospital exacerbation rate 2022					0.68	0.57	0.91	0.58	1.00
Hospital exacerbation rate 2023					0.00	0.14	0.36	0.26	0.44

**Table 2:** The spirometry data are a median value and interquartile range. The Wilcoxon signed-rank test result was used to compare values in 2022 and 2023.

	2018 *n* = 38	2019 *n* = 47	2020 *n* = 44	2021 *n* = 58	2022 *n* = 85	2023 *n* = 85	Wilcoxon Signed-Rank Test *p*-Value
**FEV1%**	57.35 (39.70–72.0)	55 (38.20–73.60)	53.70 (37.80–70.80)	54.4 (35.40–70.00)	54.00 (39.00–69.30)	63.60 (48.00–83.00)	**0.000000**
**FEV1-L**	1.91 (1.35–2.44)	1.90 (1.40–2.65)	1.87 (1.18–2.36)	1.83 (1.18–2.55)	1.79 (1.25–2.58)	2.25 (1.44–3.14)	**0.000000**
**FVC%**	71.10 (56.10–85.20)	71.30 (56.00–84.10)	65.40 (50.75–80.40)	70.50 (53.0–82.80)	69.00 (56.00–83.90)	86.10 (66.00–101.00)	**0.000000**
**FVC%**	2.69 (2.13–3.38)	2.79 (2.18–3.79)	2.60 (2.10–3.20)	2.79 (2.10–3.48)	2.64 (2.19–3.63)	3.24 (2.43–4.15)	**0.000000**

**FEV1%,** forced expiratory volume in the first second as a percentage of predictive value; **FEV1-L,** forced expiratory volume in the first second measured in litres; **FVC%,** forced vital capacity as a percentage of predictive value; **FEV1-L,** forced vital capacity measured in litres; ***n*,** number of patients; ***p*** < 0.05 is an indicator of statistical significance.

**Table 3:** The FEV1% absolute value change between 2022 (before the start of CFTR-T) and 2023 (one year after the beginning of CFTR-T) showed as median and interquartile range.

**FEV1%**	**FEV1 > 70%** ***n* = 25**		**FEV1 60–69%** ***n* = 15**		**FEV1 50–59%** ***n* = 13**		**FEV1 35–49%** ***n* = 18**	
	**2022**	92 (75.6–98.0)	**2022**	64.0 (61.0–68.0)	**2022**	54.0 (52.9–57.1)	**2022**	44.5 (39.1–48.0)
	**2023**	100.9 (81.1–110.6)	**2023**	78.4 (70.8–83.0)	**2023**	60.35 (52.0–71.1)	**2023**	50.0 (45.1–53.7)
	**Delta**	8.9	**Delta**	14.4	**Delta**	5.8	**Delta**	5.5
	**WT**	***p*** ** = 0.030**	**WT**	***p*** ** = 0.002**	**WT**	***p*** ** = 0.039**	**WT**	***p*** ** = 0.000**

**FEV1%**, forced expiratory volume in the first second as a percentage of predictive value; **delta**, the difference between median values in 2022 and 2023; **WT**, the Wilcoxon signed-rank test; ***p*** <0.05 indicates statistical significance; ***n***, number of patients.

## Data Availability

The raw data supporting the conclusions of this article will be made available by the authors on request.
